# Prevalence of Bovine Tuberculosis and Risk Factor Assessment in Cattle in Rural Livestock Areas of Govuro District in the Southeast of Mozambique

**DOI:** 10.1371/journal.pone.0091527

**Published:** 2014-03-14

**Authors:** Ivânia Moiane, Adelina Machado, Nuno Santos, André Nhambir, Osvaldo Inlamea, Jan Hattendorf, Gunilla Källenius, Jakob Zinsstag, Margarida Correia-Neves

**Affiliations:** 1 Life and Health Sciences Research Institute (ICVS), School of Health Sciences, University of Minho, Braga, Portugal; 2 ICVS/3B’s, PT Government Associate Laboratory, Braga/Guimarães, Portugal; 3 Paraclinic Department, Veterinary Faculty, Eduardo Mondlane University, Maputo, Mozambique; 4 Department of Clinical Science and Education, Södersjukhuset, Karolinska Institutet, Stockholm, Sweden; 5 Swiss Tropical and Public Health Institute, Basel, Switzerland; Fundació Institut d’Investigació en Ciències de la Salut Germans Trias i Pujol, Universitat Autònoma de Barcelona, CIBERES, Spain

## Abstract

**Background:**

Bovine tuberculosis (bTB), caused by *Mycobacterium bovis,* is an infectious disease of cattle that also affects other domestic animals, free-ranging and farmed wildlife, and also humans. In Mozambique, scattered surveys have reported a wide variation of bTB prevalence rates in cattle from different regions. Due to direct economic repercussions on livestock and indirect consequences for human health and wildlife, knowing the prevalence rates of the disease is essential to define an effective control strategy.

**Methodology/Principal findings:**

A cross-sectional study was conducted in Govuro district to determine bTB prevalence in cattle and identify associated risk factors. A representative sample of the cattle population was defined, stratified by livestock areas (n = 14). A total of 1136 cattle from 289 farmers were tested using the single comparative intradermal tuberculin test. The overall apparent prevalence was estimated at 39.6% (95% CI 36.8–42.5) using a diagnostic threshold cut-off according to the World Organization for Animal Health. bTB reactors were found in 13 livestock areas, with prevalence rates ranging from 8.1 to 65.8%. Age was the main risk factor; animals older than 4 years were more likely to be positive reactors (OR = 3.2, 95% CI: 2.2–4.7). *Landim* local breed showed a lower prevalence than crossbred animals (*Landim* × *Brahman*) (OR = 0.6, 95% CI: 0.4–0.8).

**Conclusions/Significance:**

The findings reveal an urgent need for intervention with effective, area-based, control measures in order to reduce bTB prevalence and prevent its spread to the human population. In addition to the high prevalence, population habits in Govuro, particularly the consumption of raw milk, clearly may potentiate the transmission to humans. Thus, further studies on human tuberculosis and the molecular characterization of the predominant strain lineages that cause bTB in cattle and humans are urgently required to evaluate the impact on human health in the region.

## Introduction

Bovine tuberculosis (bTB) is an infectious disease of cattle caused by *Mycobacterium bovis*, a member of the *Mycobacterium tuberculosis* complex. This chronic disease also affects a wide range of other domestic and wildlife animals and may also cause disease in humans [1].

Worldwide, bTB is considered one of the seven most neglected endemic zoonoses, presenting a complex epidemiological pattern and with the highest prevalence rates in cattle found in African countries, part of Asia and of the Americas [2]. In affected countries, the disease has an important socio-economic and public health-related impact, and represents also a serious constraint in the trade of animals and their products [3]. In developed countries, bTB was regarded as one of the major diseases of domestic animals until the 1920s [4], when preventive and control measures based on tuberculin skin test and subsequent slaughter of positive reactors and sanitary surveillance in slaughterhouses, began to be systemically applied [5]. After implementation of the control programs, bTB in cattle populations was greatly reduced or even eradicated [3]. Nevertheless, wildlife species are still considered a significant source of infection and responsible for the failure of the complete eradication of livestock bTB in some developed countries [6]. Unfortunately, a vaccination strategy for animals is not available and present bTB control strategies are expensive and difficult to implement. Consequently, in developing countries, where bTB remains of economic and public health importance [7], these strategies are often not in use or not applied systematically [1], [8]. In addition, it is estimated that in Africa 90% of the milk is consumed raw or fermented, increasing the risk of bTB transmission to humans [9].

In order to develop an effective national program for bTB surveillance and control in developing countries, accurate data on bTB prevalence is needed [10]. In Mozambique, data on bTB epidemiology is still scarce and mostly unpublished. However, bTB is estimated to be one of the most important causes of economic losses in cattle production, due to rejection of carcasses at the slaughterhouse and limitations on trade, both intra-community and between districts [11]. Surveillance and control programs based on the tuberculin skin test in cattle at the farm and subsequent slaughter of positive reactors are not applied systematically and do not cover the small holder sector due to the costs with replacement of slaughtered animals [11]. Additionally, there are no effective measures for preventing the transmission of zoonotic diseases and a “bridge” between control programs of bTB and human tuberculosis has not been implemented.

In Mozambique’s Govuro district, a great proportion of the population holds livestock animals (especially cattle and goats). According to the findings of positive skin test reactors, associated with lesions compatible with bTB found at slaughterhouses, the Provincial Livestock Services (SPP) considered Govuro positive for bTB [11] but accurate information on the bTB prevalence and its role in human tuberculosis is missing. Control measures are nowadays only based on compulsory test for bTB in cattle to be transferred for breeding or rearing purposes. While slaughter for local consumption is uncommon (only in traditional ceremonies), animals are frequently sold to be consumed in the south of the country. In Govuro there is extensive consumption of untreated milk, direct contact between people and livestock, together with malnutrition and a high prevalence rate of HIV infection, all of which constitute risk factors for this zoonosis. Also still unknown (but crucial to minimize disease propagation) are the main risk factors contributing to the spread of the disease between animals.

Previous studies conducted in Govuro in 2008, using the single intradermal tuberculin test (SITT) in the caudal fold and in the middle neck region of cattle, found prevalence values of 61.94% (n = 268) [11]. This represents the highest recorded prevalence in cattle in the country, however only two livestock areas of the district were analyzed. In order to determine the prevalence rate of bTB in Govuro we conducted a cross-sectional survey covering a representative sample of the cattle population of all livestock areas within the district. The single comparative intradermal tuberculin test (SCITT) was used since its specificity is higher than the one from the SITT [12]. While the SITT is the standard diagnostic test used in the Mozambican Bovine Tuberculosis Control Program, the SCITT is the confirmatory test and can also be used as screening test in herds with a history of cross-reactivity. We also assessed intrinsic determinants of disease associated with SCITT positivity in the study area in order to define strategies suitable to bTB control in cattle in Govuro.

## Materials and Methods

### Ethics Statement

The purpose of this study was explained to the cattle owners and an informed consent was obtained. While the SITT is the standard diagnostic test used in the Mozambican Bovine Tuberculosis Control Program we used the SCITT due to its higher specificity and to the fact that using this more complete test the data for SITT was also obtained. The Mozambican National Animal Health authority (*Direcção Nacional de Serviços Veterinários*) approved the present study and provided the ethical clearance (Nota 162/MINAG/DNSV/900/2013).

### Study Area

**Figure 1:**
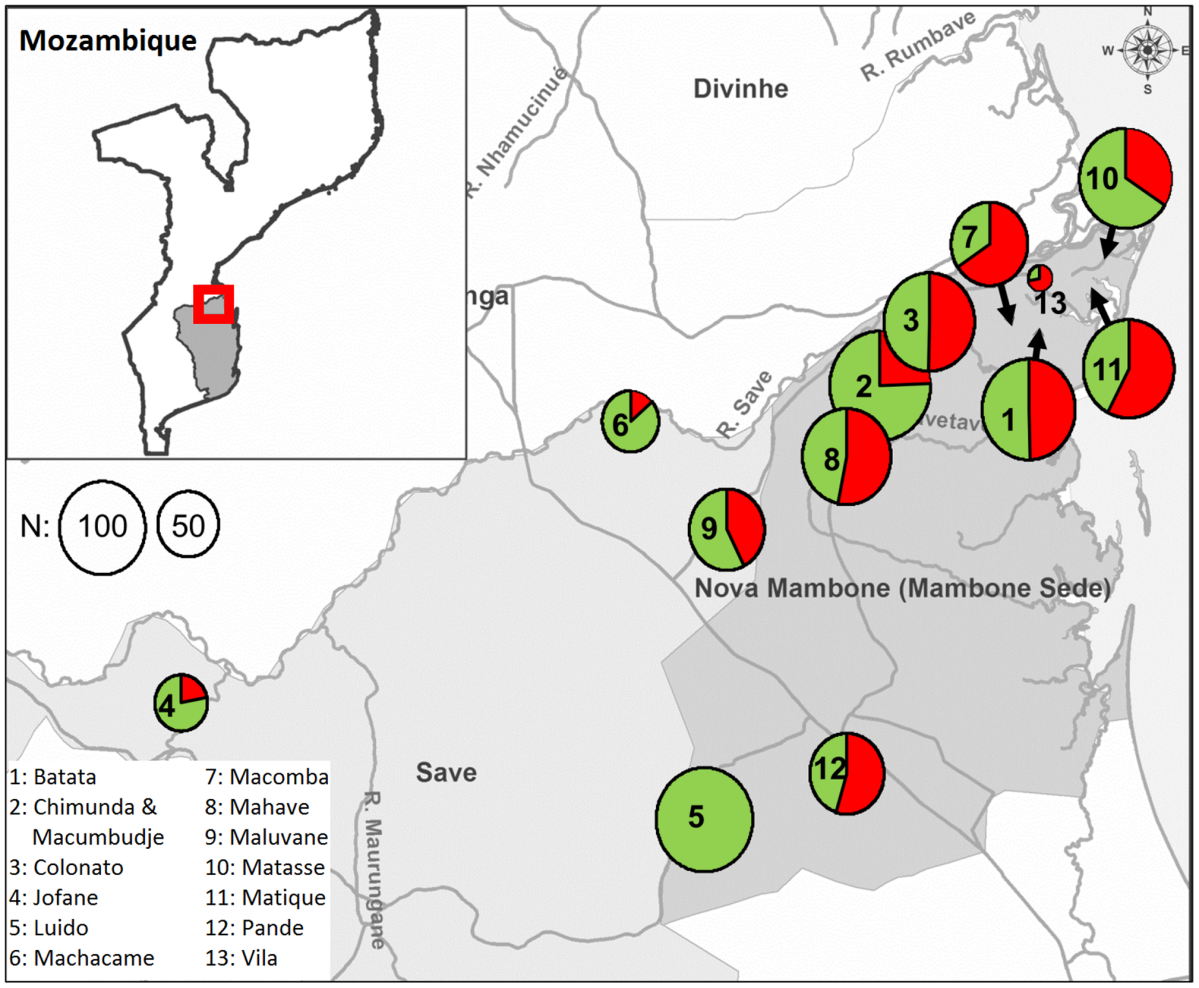
Location of the study district Govuro and spatial distribution of positive reactor cattle. The circle size is proportional to the number of animals tested in each location, and red area denotes the proportion of positive animals.

A cross-sectional study was carried out in the Govuro district (Figure 1) located in the northern part of Inhambane province, south-eastern Mozambique. The region is bordered on the north by the Machanga district of the Sofala province (across the Save River), on the east by the Indian Ocean, on the south by Inhassoro district and on the west by the Mabote district. The district covers an area of 3,960 km^2^ with an estimated population of 35,500 inhabitants. The climate is tropical dry in the interior and humid close to the coast with an average temperature of 25.5°C (18–33°C). The rainy and dry seasons generally occur around October to March and April to September, respectively [13]. Govuro comprises 2 administrative posts (Nova Mambone and Save), 5 localities, 14 livestock areas and 45 villages containing an estimated number of 773 farmers and 8,760 cattle heads.

### Animals and Production Systems Tested for Bovine TB

The animals included in this study were from the small holder sector or from the commercial sector. In the small holder sector most animals were longhorn *Landim* cattle (local breed, mixed *Bos indicus* and *Bos taurus*) and crossbreeds (*Landim* × *Brahman*) and with herds typically comprised of both these cattle breeds. Cattle were kept traditionally in a free-range grazing system using communal grazing grounds (without supplementation) and watering points such as small puddles (formed throughout the grazing area during the rainy season) or the Save River (during the dry season) [11]. Animals received little veterinary assistance, mostly restricted to vaccination. Animals from the commercial sector were mostly of *Simmental* (*Bos taurus*), *Brahman* (*Bos indicus*) and *Bonsmara* (mixed *Bos indicus* and *Bos taurus*) breeds. They were reared under semi-intensive farming with limited grazing areas (maintained in fences separated from cattle of the small holder sector) and with established water sources. Veterinary assistance and supplementation were provided. Whilst in the commercial sector the livestock production was mainly market-oriented, in the small holder farming animals were frequently used to till the ground and to transport material and people. Livestock trading in the small holder sector is restricted to special occasions, essentially when there is scarcity in agriculture production; in case of diseases and medical assistance is needed; to raise money for children’s school fees or other essential livelihood assets for the family such as food items, soap and clothes.

### Sample Size Calculation and Study Animals

To obtain a sample size representative of the Govuro district cattle population, the number of animals to be tested was calculated with Epicalc 2000 (Brixton Books v.1 2), using an expected prevalence of 10% and precision measured as one-half length of the 95% confidence interval of 5%. Sample sizes were calculated for each livestock area and corrected for finite population sizes. The epidemiological unit of this study was the livestock area, which corresponds to the cattle from several owners belonging to the same village. Even belonging to distinct owners, animals have regular direct contact between them and share natural pasture areas and watering sites (grazing groups) or even cowsheds. All cattle of the district (estimated as n = 8,763) was included in the sampling frame. The required sample sizes for the livestock areas ranged from 45 to 139 animals and resulted in a total sample size of n = 1,443 (Details are provided in Appendix S1).

All cattle owners from each livestock area were contacted to participate. The vast majority of the owners brought their animals to pre-defined locations. For the owners that could not bring the animals to the testing place we went to their home place. As a result more than 6,000 cattle participated, out of an estimated population of 8,760. Animals were selected randomly by systematic sampling according to the sample size previously determined and the number of cattle present on the day of the test. They were moved through a cattle chute and every k^th^ animal was selected for sampling, being k the number of animals presented for testing in that livestock area divided by the intended sample size for that same livestock area. At the time of SCITT testing, each animal was identified by a numbered ear tag and individual animal data on age, gender, breed, body condition score (BCS) and owner were registered. Information regarding the age of the animals was provided by the farmers and the breed was determined according to the phenotypic characteristics. The body condition was scored using the guidelines established by Nicholson and Butterworth [14]; all study animals were categorized in four groups: very poor (1), poor (2 to 3), reasonable (4 to 6) and good (score, 7 to 9).

### Single Comparative Intradermal Tuberculin Test

The purpose of the study was explained to the owners with the assistance of local veterinary services (SDAE), community leaders, the local prosecutor and trusted intermediaries. SCITT was performed by intradermal injections of both avian and bovine purified protein derivates (PPD) in the middle neck region (usually on the right side) according to the method described by the World Organization for Animal Health standards [2]. Briefly, two sites of about 2 cm^2^ diameter, approximately 12 to 15 cm apart, were shaved and the skin thickness was measured using a manual caliper. Aliquots of 0.1 ml containing 20,000 IU/ml of bovine PPD (Bovituber PPD, Synbiotics Europe, Lyon, France) and 0.1 ml with 25,000 IU/ml of avian PPD (Avituber PPD, Synbiotics Europe, Lyon, France) were injected using two different syringes into the dermis in the corresponding shaved area. Palpation of a small grain-like thickening at each site of injection was done to confirm the correct intradermal injection. Three days after injection, the tested animals were brought back for reading. The relative change in skin appearance was classified as swelling or induration followed by measurement of skin thickness at both injection sites. Skin thickness measurements on testing and reading day were performed by the same person to avoid errors related to individual variations in technical procedure.

The SCITT results were analyzed and interpreted according to the recommendations of the World Organization for Animal Health standards [2]. The reaction was considered positive if the increase in skin thickness at the bovine PPD site of injection *(B_72_-B_0_)* was at least 4 mm greater than the reaction at the avian PPD injection site *(A_72_-A_0_)*. The livestock area was considered positive for bTB if at least one positive reactor was found.

Additionally we determined the SITT results by analyzing the same dataset taking into consideration only the bovine PPD data, using the same cutoff. Also, to assess the prevalence of reactors to other sensitising organisms such as *Mycobacterium avium*, the skin reactions at the injection site of the avian PPD alone were analyzed; animals that reacted to the avian PPD with an increase in skin thickness equal or superior to 4 mm were considered reactors to *M. avium*. Geographical coordinates were registered at the central point of each livestock area by a hand held global positioning system.

### Data Analysis

All data at individual animal level were entered into a Microsoft Access database. Data analysis was performed in R statistical software (v2.15.1). Prevalence, odds ratios (OR) and their 95% confidence intervals were adjusted for correlation within livestock areas using generalized linear mixed models with binary outcome and livestock area as random effect.

## Results

### Sample Characteristics

Over the study period a total of 1,419 cattle were injected with PPDs and measurements were obtained from 1,136 animals (80%) belonging to 289 farmers. Table 1 shows the main characteristics of the sample tested. One hundred and twenty five (11%) animals came from the commercial sector (all from the Luido area) and 1,011 (89%) from the small holder sector. About two third of the cattle were female. The age distribution was as follows: 3% of the animals between 0 to 1 years old (calf - “<1 year”); 22% between 1 to 4 years old (steer - “1–4 years”); and 75% were older than 4 years (bull, cow and ox - “>4 years”). Almost half (49%) of the animals were of crossbreeds (*Landim* × *Brahman*), 43% *Landim* and 8% *Bonsmara*. *Simmental*, *Brahman* and *Limousine* were tested only in one herd from the commercial sector in Luido representing 1% of the sample. Sixty-two percent of all animals tested were classified as having good BCS, 30% reasonable BCS, and a small proportion presented poor (8%) and very poor (0.2%) BCS. Characteristic measures were not recorded for a few animals (Table 1).

**Table 1:** Basic characteristics of the sample.

Characteristics	Classes	n	%
Gender	Female	773	68.0
	Male	362	31.9
	Not recorded	1	0.1
Age	0–1 yr (calf)	38	3.3
	1–4 yrs (steer)	245	21.6
	>4 yrs (bull, cow, ox)	852	75.0
	Not recorded	1	0.1
Breed	*Landim*	468	41.2
	Crossbred (L*andim* × *Brahman*)	534	47.0
	*Bonsmara*	88	7.7
	Other	11	1.0
	Not recorded	35	3.1
Body condition score	Good	705	62.1
	Reasonable	337	29.7
	Poor	86	7.6
	Very poor	2	0.2
	Not recorded	6	0.5

### Cattle bTB Prevalence in Govuro

The results of the SCITT are presented in Table 2 as prevalence per livestock area. The overall apparent prevalence of SCITT positive reactors was 39.6% (95% CI: 36.8–42.5). Except in Vila, where most of the PPD-inoculated animals failed the reading day, representative samples were obtained for each of the other 13 livestock areas. Among them, only in Luido, where the animals were all from the commercial sector, no SCITT positive reactors were detected (Table 2). In addition, data shows that bTB prevalence rates vary remarkably between livestock areas (ranging from undetectable up to 65.8%).

**Table 2:** Apparent prevalence of bTB in Govuro per livestock area.

Livestock area	Animals tested		Bovine PPD SCITT reactors			Bovine PPD SITT reactors			Avian PPD SITT reactors		
	Total	Read	n	%	95% CI	n	%	95% CI	n	%	95% CI
Batata	133	117	58	49.6	40.7–58.5	78	66.7	57.7–74.6	17	14.5	9.3–22.0
Macomba	101	79	52	65.8	54.9–75.3	60	75.9	65.5–84.0	7	8.9	4.4–17.2
Colonato	173	111	56	50.5	42.3–59.6	70	63.1	53.8–71.5	12	10.8	6.3–18.0
Jofane	37	37	8	21.6	11.4–37.2	11	29.7	17.5–45.8	1	2.7	0.5–13.8
Maluvane	84	76	32	42.1	31.7–53.3	39	51.3	40.3–62.2	4	5.3	2.1–12.8
Matasse	123	115	39	33.9	25.9–43.0	53	46.1	37.2–55.2	16	13.9	8.6–21.4
Chimunda	75	62	27	43.5	31.9–56.0	37	59.7	47.2–71.0	16	25.8	16.6–37.9
Mahave	150	105	56	53.3	43.8–62.6	74	70.5	61.2–78.4	15	14.3	8.9–22.2
Matique	142	111	64	57.7	48.4–66.4	83	74.8	66.0–82.0	22	19.8	13.5–28.2
Pande	95	74	41	55.4	44.1–66.2	46	62.2	50.8–72.4	8	10.8	5.6–19.9
Luido	130	125	0	0.0	0.0–3.0	1	0.8	0.1–4.4	8	6.4	3.3–12.1
Vila	43	7	5	71.4	35.9–91.8	5	71.4	35.9–91.8	0	0.0	0.0–35.4
Machacame	45	43	6	14.0	6.6–27.3	9	20.9	11.4–35.2	3	7.0	2.4–18.6
Mucumbudje	90	74	6	8.1	3.8–16.6	20	27.0	18.2–38.1	8	10.8	5.6–19.9
Total	1421	1136	450	39.6	36.8–42.5	586	51.6	48.7–54.5	137	12.1	10.3–14.1

SCITT results showed that 137 (12%; 95% CI: 10.3–14.1) out of 1,136 cattle tested were positive reactors to avian PPD (Table 2). Among the 137 cattle with a positive reaction to avian PPD, 49 (36%; 95% CI: 28.2–44.1) had an overall SCITT test also positive but 24 (18%; 95% CI: 12.1–24.8) showed a stronger response to avian PPD than to bovine PPD.

### Risk Factors Associated with Positive Reaction to SCITT

**Figure 2:**
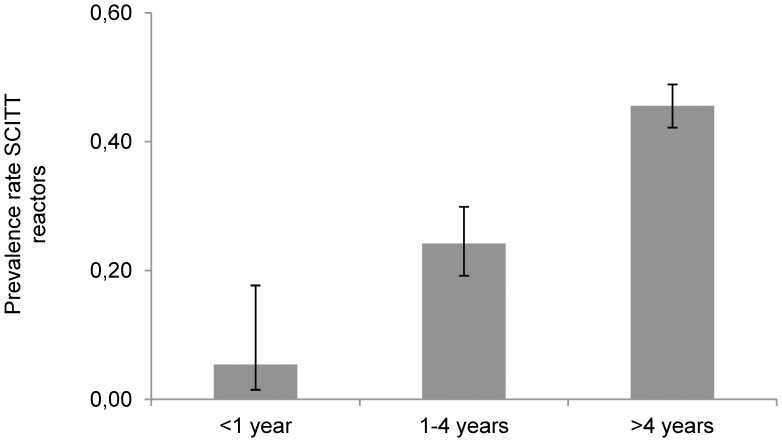
Prevalence rates among SICTT reactors (95% confidence intervals) by age classes.

Univariate and multivariate analysis showed that age and breed represented intrinsic risk factors associated with positive reaction to SCITT (Table 3). Animals older than 4 years were more likely to be infected compared to young animals (45.4% *vs* 21.9%; OR = 3.2, 95% CI 2.2–4.7) (Table 3), and this difference was statistically significant even when considering the age classes “<1 year”, “1–4 years” and “>4 years” (χ^2^ = 55.56; d.f. = 2, P<0.001) (Figure 2). Male animals tended to show higher prevalence rates for bTB (42.7% *vs* 37.2% in females), but there was no statistically significant difference in reactivity to the SCITT test between gender (χ^2^ = 2.20; d.f. = 1; P>0.05).

**Table 3:** Risk factors associated with positive reaction to SCITT.

Risk factor	Category	Positive/total	Positive (%)	Univariate analysis		Multivariate analysis		
				OR	95% CI	OR	95% CI	p
Age	≤4 yrs	62/283	22	reference		reference		
	>4 yrs	388/854	45	2.9	2.0–4.1	3.2	2.2–4.7	<0.001
Gender	Female	259/773	38	reference		reference		
	Male	155/362	43	1.1	0.8–1.4	1.2	0.9–1.6	0.300
Breed	*Landim* × *Brahman*	265/534	50	reference		reference		
	*Landim*	167/468	36	0.7	0.5–0.9	0.6	0.4–0.8	0.002
	*Bonsmara* a	0/88	0	nd		nd		
Body condition score	Good	250/705	35	reference		reference		
	Reasonable	153/337	45	1.0	0.8–1.4	1.0	0.7–1.4	0.820
	Poor and Very poor	44/88	50	1.4	0.8–2.3	1.3	0.7–2.1	0.410

a Excluded from the multivariate model to avoid quasi separation.

The rates of SCITT bTB reactors were higher in crossbreeds (*Landim* × *Brahman*) when compared to local *Landim* breed. Out of 468 of the *Landim* breed animals tested, 167 were found to be positive for the disease (35.7%; 95% CI 31.5–40.1) whereas 265 out of 534 (49.6%; 95% CI 45.4–53.9) in the crossbred (*Landim* × *Brahman*) cattle were positive reactors (Table 3). These data revealed a statistically significant association between the type of breeds and bTB prevalence, where the *Landim* breed seemed to be at lower risk for infection (OR = 0.6; 95% CI 0.4–0.8). All animals of the breed *Bonsmara* belonged to two private farmers from the livestock area Luido, where no positive reactors were found (0/88).

## Discussion

Our results show that bTB is highly prevalent in Govuro district, with an overall prevalence rate of 39.6%. The sample size in each livestock area was slightly lower than targeted and the observed prevalence was closer to 50%, consequently, the precision associated with the prevalence estimates for the single livestock areas was lower than planned in the sample size calculation. However, the overall prevalence and risk factors were associated with high precision and narrow interval estimates. The SCITT has a less than perfect sensitivity, with a range of 52.0–95.5%, dependent on local factors [12]. Adjusting for the relatively low sensitivity of the SCITT, we estimated that the true prevalence in Govuro district is likely to be substantially higher than the apparent prevalence. The Rogan-Gladen estimator yielded a true prevalence of 65%, assuming a test specificity of 0.96 and sensitivity of 0.59, recalculated from data on Chadian cattle [15].

A high prevalence of bTB was observed in almost all livestock areas where small scale farming was practiced, in sharp contrast with what was observed in the commercial sector (only present in Luido), where no SCITT positive animals were detected. While in the commercial sector animals are normally tested for bTB and kept in quarantine before being introduced, trading of animals among breeders in the small holder sector is frequently performed without previous information about bTB status of the animals. In addition, the two tested farms in Luido were established in 2008, only four years before sampling. Interestingly, in the two livestock areas with the lowest bTB prevalence in the small holder sector (Mucumbudje and Machacame), livestock was just recently introduced (years 2007–2008). Our data show that age of the animals was an important intrinsic risk factor, most probably associated with increased exposure to *M. bovis* with lifetime. The type of management system applied in the small holder sector in Govuro, with sharing of water points and grazing areas, and close contact between animals from the same or different herds, promotes the spread of respiratory diseases such as bTB [4], [16], [17]. Additionally, during vaccination campaigns or external deworming, the animals from different farmers or herds use the same dip tanks. In contrast, animals from the commercial sector are kept inside fences and reared on a rotational grazing system with no contact with cattle from the small holder sector.

A study carried out in 2008 in the same region reported a bTB prevalence rate of 61.9% (95% CI: 55.8–67.8) [11]. Together with the present study, these data suggests that bTB is stable at an extremely high prevalence in the region. In the previous study the covered sample was limited to two livestock areas (Colonato and Vila), whereas in the present study all livestock areas were included. In addition, this study by Macucule et al. [11] made use of the single intradermal tuberculin test (SITT) while in the present study we made use of the SCITT. When our data were analyzed taking in consideration only the bovine PPD result, which corresponds to the SITT, the prevalence rates obtained (63.6%, 95% CI: 0.55–0.72) were similar to what was reported from these two livestock areas in 2008.

The choice of the SCITT instead of the SITT has been shown to be of relevance to differentiate between animals infected with *M. bovis* and those responding to bovine PPD possibly as a result of exposure to other mycobacteria. In fact, in our study the overall prevalence of bTB, taking into consideration only the bovine PPD results, was 51.6% (95% CI: 48.7–54.5), clearly higher than the one determined using the SCITT. This higher rate of positive SITT reactors can be attributed to sensitization with cross-reactive antigens among mycobacterial species and related genera [2].

According to the definitions of positivity, the animals that reacted equally to both PPDs (avian and bovine) were classified as negative reactors to SCITT [2]. Reactivity to the avian PPD may indicate infection or simply exposure to species of the *M. avium* complex or other environmental mycobacteria. This reactivity, however, may indicate a mixed reaction to both agents and hence the classification of bTB negative might also lead to some false negatives. The equal reactivity in both sites of injections (avian and bovine PPD) could be related with a generalized sensitization in which the immune response is not specific to a particular mycobacteria species.

In our study we found 137 (12%) animals that reacted positively to the avian PPD, a finding that has also been previously described. In a cross-sectional study done in Uganda, Inangolet *et al*. [18] attributed the high number of avian reactors in cattle with the existence of large poultry population in the studied areas, where chicken production in a free-ranging system is common. Fecal contamination of the watering sources was indicated as the main route of transmission of *M. avium* to cattle. In our study area, poultry production is a common activity, nevertheless the system where cattle are kept in corrals (mainly during the night), away from residences, do not promote direct and frequent contact between these two species. The reactivity in the avian PPD in Govuro could be associated with the high population density of cattle egret (*Bubulcus ibis*) in the district. This species is usually found along grazing cattle (removing ticks and flies from the animals). In fact, the presence of *M. avium* subsp. *avium* was already found in fecal samples of cattle egret [19] which could constitute a source of spread to the cattle.

The causative agent of avian tuberculosis, *M. avium* subsp. *avium,* was the predominant MAC isolated from tuberculous lesion in cattle [20]. The role of small ruminants (goats and sheep) as vector of *M. avium* subsp. *avium* and *Mycobacterium avium* subsp. *paratuberculosis* has also been identified [3]. In Govuro, the predominant production system is the communal/pastoral system, where the small ruminants graze together with cattle. Sharing of pastures and watering points could represent a potential source of infection of *M. avium* to cattle. However, according to Okuni et al. [21], paratuberculosis in cattle was not reported in Mozambique. Further studies are necessary to clarify the source of the avian PPD reactions found in cattle in Govuro.

In accordance with findings from numerous cross-sectional studies conducted in both developed and developing countries [e.g. 18,22–25], our results show that age was the main individual risk factor. Some authors suggest that it could be related to increased duration of exposure with age, with older cattle being more likely to have been exposed than the younger [24], [26]. Out of 38 calves tested only 2 (5.26%) had a positive result on SCITT. The low number of positive cases in young animals may be associated with the predominance of gamma delta (γδ) T cells in calves that have been shown to play a relevant role in antimycobacterial immunity [27]. The positive calves (although in low number) could be due to congenital transmission in utero [28]. In addition, ingestion of contaminated colostrum has already been reported as another route of bTB transmission [29], as well as pseudo-vertical transmission (close contact between cow and its calf) [30].

The analysis of our bTB reactors according to gender showed that, although the reactivity among males was slightly higher (43% *versus* 38%) the difference was not statistically significant. Male cattle were identified as being the group at highest risk in other studies due to their particular longevity in the herd, given their use as draught oxen, facilitating maintenance of the infection in the herds [22]. Higher reactivity of females than males was previously reported in dairy cows [18], [25] and associated with their maintenance in the same herd for several years [5]. In Govuro, however, male cattle tend to be maintained for longer periods in the herds since they are commonly used for plowing the land and pull carts for transportation of people and goods.

Most of the cattle included in the present study were crossbreed (*Landim* × *Brahman*) and *Landim* local breed and bTB prevalence rates were found to be significantly higher in this later breed. The cattle recorded as *Simmental* and *Bonsmara* breed, from the two commercial sector farms in Luido, were too few to allow a relevant comparison of susceptibility. Several studies [31], [32] have shown a variation in susceptibility to bTB among cattle breeds, with European breeds (*Bos taurus*) being less resistant compared to Zebu cattle (*Bos indicus*). Although crossbred cattle in Ethiopia (local *Bos indicus* breed Arsi × Holstein *Bos taurus* breed) has been suggested to exhibit intermediate levels of susceptibility [31], our data does not support this observation, as animals with more Zebu background (*Landim* × *Brahman*) showed higher bTB prevalence than *Landim* cattle. It should be mentioned that differences in bTB prevalence between breeds - as observed in several studies - can be influenced by different husbandry conditions; however, genetic variations among cattle breeds are also likely to have an influence on susceptibility to infection with *M. bovis*. The genetic variations among cattle breeds have an influence on susceptibility to infection with *M. bovis*. In diverse breeds of British cattle, the genomic regions INRA111 and BMS2753 were strongly associated with bTB infection status [33]. Two others loci have also been linked to susceptibility in Holstein cattle, namely, a variant in the TLR1 gene [34] and BTA 22 [35].

Several studies reported a correlation between body condition and bTB [e.g. 10,36]. In our study animals in reasonable and poor or very poor body condition showed more positive skin test results than animals in good body condition, however this difference did not reach statistical significance. Following recommendations by Humblet *et al*. [5], this parameter should be analyzed carefully, since while a poor BCS might be a cause of disease, it is also extremely influenced by the seasonal climatic changes (rain or dry season) and the consequently more or less availability of pasturage and/or prevalence of intestinal parasites (in the small scale small holder farming of Govuro deworming for internal parasites is uncommon). It was reported previously [10], [36] that animals in very poor body condition could be non-responsive to the SCITT due to anergy caused by immune-suppression. Our results do not support this finding. In addition, in cross-sectional studies, the status of the animal before becoming infected is not known, and thereby it is impossible to distinguish if the poor body condition was a risk factor or if it is a consequence of advanced stage of bTB.

This is the first systematic study on bTB prevalence encompassing a representative sampling of all livestock areas of a particular district in Mozambique. The data clearly show that bTB is a serious problem in Govuro district with extremely high prevalence rates being maintained for several years. Our results strengthen the notion that if strong measures were undertaken, as was the case among the commercial sector, the disease might be controlled. It is of relevance to stress that drinking raw milk is a common habit in Mozambique, especially for young children that take in charge the livestock grazing. In addition, due to their stature, children that graze animals may be extremely exposed to *M. bovis* airborne transmission from infected animals. Taking all this into account and the fact that studies on human tuberculosis have not been systematically performed in Govuro (neither for *M. tuberculosis* nor *M. bovis*) our results reinforce the need not just to undertake bTB control measures in the region but also the urgency to investigate the prevalence of tuberculosis in humans, especially in children in Govuro.

## Supporting Information

Appendix S1 **Sample Size Calculation.**
(DOCX)
