# Supplementary material for: Prevalence of Bovine Tuberculosis and Risk Factor Assessment in Cattle in Rural Livestock Areas of Govuro District in the Southeast of Mozambique
Source: PLoS One. 2014 Mar 14;9(3):e91527. doi: 10.1371/journal.pone.0091527 (PMC3954769; doi:10.1371/journal.pone.0091527)
Supplement: Appendix S1 — Sample Size Calculation. (DOCX) [file pone.0091527.s001.docx]

**Supplementary data appendix – Sample size calculation.**

The number of animals to be tested was calculated with Epicalc 2000 (Brixton Books v.1 2), using an expected prevalence of 10% and precision measured as one-half length of the 95% confidence interval of 5%. Sample sizes were calculated for each livestock area and corrected for finite population sizes.

| **Livestock area** | **N. of cattle** | **N. of cattle to be tested according Epicalc 2000** | **20% of sample size (expected non-compliance)** | **N. of animals to be tested + 20%** |
| --- | --- | --- | --- | --- |
| Batata | 721 | 128 | 26 | 154 |
| Macomba | 653 | 126 | 25 | 151 |
| Colonato | 1435 | 139 | 28 | 167 |
| Jofane | 97 | 63 | 13 | 76 |
| Maluvane | 153 | 80 | 16 | 96 |
| Matasse | 876 | 131 | 26 | 157 |
| Chimunda & Mucumbudje^1^ | 992 | 134 | 27 | 161 |
| Mahave | 1006 | 134 | 27 | 161 |
| Matique | 1014 | 134 | 27 | 161 |
| Pande | 527 | 121 | 24 | 145 |
| Luido | 501 | 80 | 16 | 96 |
| Vila | 730 | 128 | 26 | 154 |
| Machacame | 58 | 45 | 9 | 54 |
| **Total** | **8.763** | **1.443** | **290** | **1.733** |

^1^ Only aggregated values for number of cattle were available for these two neighboring livestock areas.
